# The Effect of a Mediterranean Diet on Arterial Stiffness: A Systematic Review

**DOI:** 10.3390/nu17071192

**Published:** 2025-03-28

**Authors:** Roberta Zupo, Fabio Castellana, Giuseppe Lisco, Filomena Corbo, Pasquale Crupi, Rodolfo Sardone, Feliciana Catino, Simone Perna, Loreto Gesualdo, Madia Lozupone, Francesco Panza, Maria Lisa Clodoveo

**Affiliations:** 1 Department of Interdisciplinary Medicine (DIM), University of Bari Aldo Moro, Piazza Giulio Cesare 11, 70100 Bari, Italy; roberta.zupo@uniba.it (R.Z.); fabio.castellana@uniba.it (F.C.); giuseppe.lisco@uniba.it (G.L.); 2 Department of Pharmacy-Drug Sciences, University of Bari Aldo Moro, 70125 Bari, Italy; filomena.corbo@uniba.it; 3 Department of Agricultural, Food and Forest Science, University of Palermo, Viale delle Scienze, 90128 Palermo, Italy; pasquale.crupi@unipa.it; 4 Urban Health Center-Local Health Authority of Taranto, 74123 Taranto, Italy; rodolfo.sardone@asl.taranto.it (R.S.); feliciana.catino@comune.taranto.it (F.C.); 5 Department of Eye and Vision Sciences, University of Liverpool, Liverpool L69 7ZX, UK; 6 Department of Food, Environmental and Nutritional Sciences, Division of Human Nutrition, University of Milan, 20133 Milan, Italy; simone.perna@unimi.it; 7 Department of Emergency and Organ Transplantation, University of Bari Aldo Moro, 70121 Bari, Italy; loreto.gesualdo@uniba.it; 8 Neurodegenerative Disease Unit, Department of Basic Medicine, Neuroscience, and Sense Organs, University of Bari Aldo Moro, 70121 Bari, Italy; madia.lozupone@gmail.com; 9 Department of Interdisciplinary Medicine (DIM), “Cesare Frugoni” Internal and Geriatric Medicine and Memory Unit, University of Bari Aldo Moro, Piazza Giulio Cesare 11, 70100 Bari, Italy; f_panza@hotmail.com

**Keywords:** arterial stiffness, Mediterranean diet, cardiovascular risk, systematic review

## Abstract

**Background:** The Mediterranean diet has long been associated with better cardiovascular health, with evidence suggesting that it may play a key role in reducing arterial stiffness. This research aims to systematically review existing evidence on the association between a Mediterranean diet pattern and arterial stiffness in the general population. **Methods**: The literature was examined in six electronic databases up until December 2024. The evaluation of the 128 publications based on inclusion criteria resulted in the selection of 16 observational and randomized controlled trials that aligned with the research question. Two researchers simultaneously extracted the data, employing inter-rater reliability (IRR) to assess coder agreement, followed by the κ statistic to evaluate accuracy and precision. According to the PRISMA principles and quality evaluation procedures, all data extraction phases achieved a k coefficient of no less than 0.9. All publications, with the exception of randomized controlled trials (RCTs), were evaluated for bias risk utilizing the NIH Quality Assessment Toolkit. The study protocol was registered with PROSPERO (CRD42024597173). **Results**: Most studies were observational (ten cross-sectional, three longitudinal), with three RCTs. Studies were primarily conducted in Europe (82%), followed by America (12%) and Australia (6%), with a total of 13,680 participants. The evidence showed an inverse relationship between adherence to the Mediterranean diet and arterial stiffness, with a focus on pulse wave velocity (PWV) and the Augmentation Index (AIx) as outcome measures. Lower but consistent and statistically significant evidence was also found in the cross-tabulation of adherence to the Mediterranean diet and the cardiovascular ankle index (CAVI), a proxy of the overall stiffness of the artery from the origin of the aorta to the ankle. Study quality ranged from moderate to high. **Conclusions**: The available evidence consistently shows that people who follow a Mediterranean diet may have less stiff arteries and, therefore, a lower cardiovascular risk. However, multifactorial biological pathways still need to be corroborated.

## 1. Introduction

Comprehensive research has been conducted on the beneficial effects of the Mediterranean diet on cardiovascular health, with a particular emphasis on the prevention and management of chronic diseases, including cardiovascular disease (CVD) and type 2 diabetes. As for diabetes, the Mediterranean diet enhances insulin sensitivity and regulates blood sugar levels, thus reducing the risk of type 2 diabetes. Its high fiber content, from plant-based foods, also aids in better glucose control. This diet pattern is also linked to improved cardiovascular health by reducing blood pressure, lowering LDL cholesterol, and improving blood vessel function, as well as arterial stiffness, a key marker of vascular health and cardiovascular risk [1].

The Mediterranean diet, characterized by a substantial consumption of fruits, vegetables, whole grains, seafood, and healthy fats such as olive oil, has been proposed as an effective dietary approach to improve these parameters [2,3]. It is a nutritional pattern inspired by the traditional eating habits of countries bordering the Mediterranean Sea. It emphasizes a high intake of plant-based foods such as fruits, vegetables, whole grains, legumes, and nuts while using olive oil as the primary fat source. Fish and seafood are consumed frequently, while red meat and processed foods are limited. This diet is rich in monounsaturated fats, omega-3 fatty acids, fiber, and antioxidants, promoting heart health, reducing inflammation, and lowering the risk of chronic diseases like cardiovascular disease, diabetes, and certain cancers. The Mediterranean diet has been widely studied for its association with improved longevity and overall well-being.

Recent studies indicate that long-term adherence to a Mediterranean diet correlates with substantial reductions in cardiovascular mortality and improvements in biomarkers of vascular health, including adipokines and ceramides, which serve as indicators of inflammation and plaque susceptibility [4,5]. Adherence to a Mediterranean diet may also lead to reduced arterial stiffness, indicating that its nutritional elements may provide protective benefits against endothelial dysfunction and inflammation, both critical contributors to cardiovascular disease [6,7]. Indeed, considering pulse wave velocity (PWV) and Augmentation Index (AIx) as key markers of arterial stiffness, a higher PWV may increase cardiac workload, blood pressure, and strain on the heart, contributing to hypertension, heart failure, and stroke. On the other hand, higher AIx values, reflecting pressure wave reflection, may increase arterial stiffness and CVD risk. Both PWV and AIx correlate with risk factors like age, hypertension, diabetes, and dyslipidemia, serving as important markers for early detection and management of CVD.

The current literature provides a number of pieces of evidence on the effect of the Mediterranean diet on arterial stiffness through clinical trials and observational studies. Literature indicates that adopting this diet pattern would improve cardiovascular health metrics and reduce the risk of chronic diseases such as diabetes and hypertension [8,9,10]. Particularly, the anti-inflammatory properties of foods like olive oil and fatty fish help maintain endothelial integrity, reduce arterial stiffness, and improve overall cardiovascular function.

Therefore, this research aims to provide a systematic review of existing evidence surrounding the impact of the Mediterranean diet on arterial stiffness and its subsequent role in the prevention of cardiovascular disease.

## 2. Methods

### 2.1. Search Strategy and Data Extraction

The present systematic review was compiled using the PRISMA 27-item checklist and the Preferred Reporting Items for Systematic Reviews and Meta-Analyses (PRISMA) criteria [11]. An a priori protocol for the search strategy and inclusion criteria was devised and registered on PROSPERO, a prospective international register of systematic reviews (CRD42024597173), without altering the information supplied at registration. We performed independent searches in the US National Library of Medicine (PubMed), Medical Literature Analysis and Retrieval System Online (MEDLINE), EMBASE, Scopus, Ovid, and Google Scholar databases to identify original research investigating the correlation between exposure to a Mediterranean diet and markers of arterial stiffness. Therefore, the main objective was to evaluate the association between exposure to a Mediterranean dietary pattern (evaluated by means of adherence scores or dietary patterns) and well-recognized markers of arterial stiffness, including aortic pulse wave velocity (a-PWv), carotid-femoral pulse wave velocity (cfPWV), brachial-ankle pulse wave velocity (baPWV), augmentation index (AIx, %) in the brachial artery, the distensibility coefficient (DC, a measurement of the elastic properties of arteries), and the cardio-ankle vascular index (CAVI). During the research selection process, we also took into account the gray literature by accessing the biggest preprint repository, https://arxiv.org/, and the http://www.opengrey.eu/ (accessed on 31 December 2024) database to obtain conference abstracts and other non-peer-reviewed content. The search approach adhered to PECO (Populations, Exposure, Comparator, and Outcomes) concepts [12], which comprise populations (humans), exposure (Mediterranean diet), comparators (exposure levels), and outcome (arterial stiffness), as we focused exclusively on observational and RCT studies. 

Table 1 describes the search approach that was used for PubMed and MEDLINE and modified for the remaining four electronic sources. There was no time limit on the literature search, and papers were found up until 31 December 2024. No linguistic limitations were introduced. Following an exhaustive literature review, two researchers (RZ, FC) independently assessed the titles and abstracts of the identified publications, verified the complete texts, and chose a limited number for inclusion in the study. Letters to the editor, technical reports, and publications from systematic and narrative reviews were excluded. The degree of agreement between coders was measured using inter-rater reliability (IRR), and accuracy and precision were then evaluated using the κ statistic. A coefficient k of at least 0.9 was achieved in all data extraction stages, according to PRISMA ideas and quality evaluation procedures [13].

### 2.2. Inclusion Criteria, Data Extraction, and Registration

No criteria were used to determine the study population’s recruiting context or health status. Potentially eligible papers were found by evaluating their abstracts and, if applicable, the full-text versions. A third researcher (MLC) reviewed the data and resolved any differences that arose. In a piloted format, the subsequent information was extracted by two investigators (RZ, FC) in duplicate: (1) General information regarding individual studies (author, year of publication, country, age, sex, design, sample size); (2) Exclusion criteria for the study population; (3) Exposure (Mediterranean diet, as evaluated by questionnaires, indices, or adherence scales); (4) Outcome; (5) Strength of the association.

A skilled biostatistician (FC) managed the MS Excel software platform for data gathering, selecting all references for retrieval from databases. Finally, the data extracted from selected studies and recorded in the database were organized as evidence tables.

### 2.3. Risk of Bias Across Studies

The included studies’ methodological quality was independently rated by paired investigators (RZ, FC) utilizing the National Institutes of Health Quality Assessment Toolkits for Observational Cohort and Cross-Sectional Studies [14,15]. Studies were assigned ratings of high (excellent), fair (moderate), or poor based on the primary toolkit criteria. This instrument comprises 14 inquiries that assess bias risk, type I and type II errors, transparency, and confounding variables, including study question, population, participation rate, inclusion criteria, justification of sample size, timing of exposure/outcome measurement, duration, exposure levels, defined exposure, blinded assessors, repeated exposure, defined outcomes, attrition, and confounding factors. Items 6, 7, and 13 do not pertain to cross-sectional research, with the maximum achievable scores for cross-sectional and prospective studies being 8 and 14, respectively. The two investigators debated the methodological quality of the papers in the review until they reached a consensus, aided by a third investigator (MLC).

## 3. Results

The initial systematic literature search yielded 128 entries. A total of 37 documents were identified as potentially relevant and selected for abstract and title analysis after the elimination of duplicates. Subsequently, 21 participants were excluded due to poor alignment with the research question. The final qualitative and quantitative analysis included only 16 papers that met the inclusion criteria after the full text of the remaining papers was reviewed [16,17,18,19,20,21,22,23,24,25,26,27,28,29,30,31].

**Figure 1:**
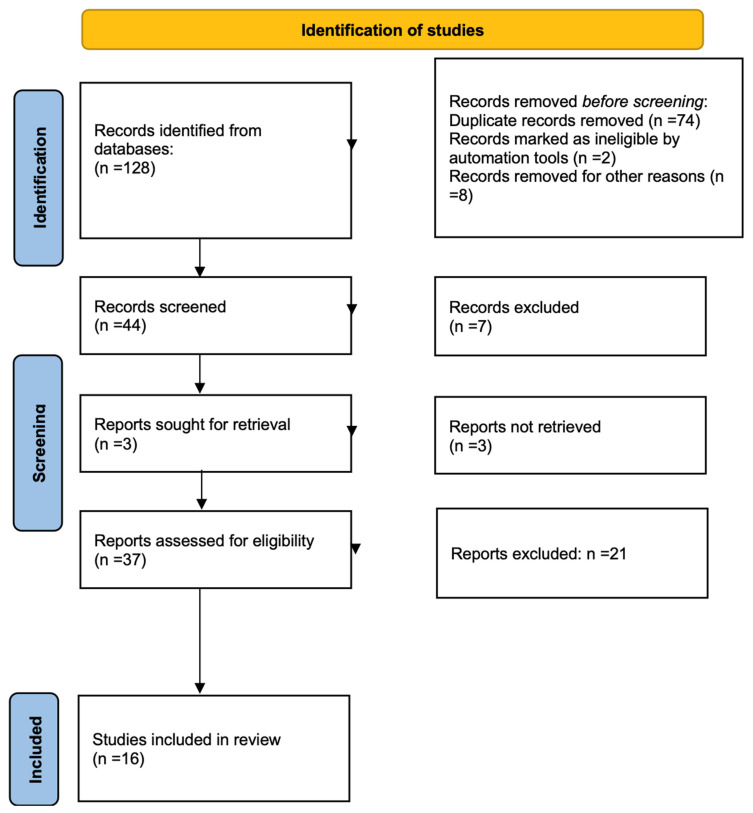
PRISMA flowchart of the screening process.

The number of studies in each stage of the review is illustrated in the flow chart of the Preferred Reporting Items for Systematic Reviews and Meta-analyses (PRISMA) in Figure 1. A total of 16 original articles were included in the final study base, which comprised both observational and RCT studies that investigated the correlation between arterial rigidity in the general population and exposure to a Mediterranean diet. 

Table 2 shows descriptive data extracted and related to study design, population age, sample size (*N*), population type, country, exclusion criteria for the study population, exposure assessment tool (i.e., Mediterranean diet adherence score, indices, or scales), outcome (i.e., arterial stiffness) assessment tool(s), and strength of the association. Most studies featured an observational design (*N* = 10 cross-sectional, *N* = 3 longitudinal) with a minority of RCTs (*N = 3*). The geographic distribution of studies was dominated by Europe (82%, *N* = 13), with a minority from America (12%, *N* = 2) and Australia (6%, *N* = 1). The total population included 13,680 individuals, mostly adults (18+ years of age), except for three studies involving children and adolescents [21,24,29].

**Figure 2:**
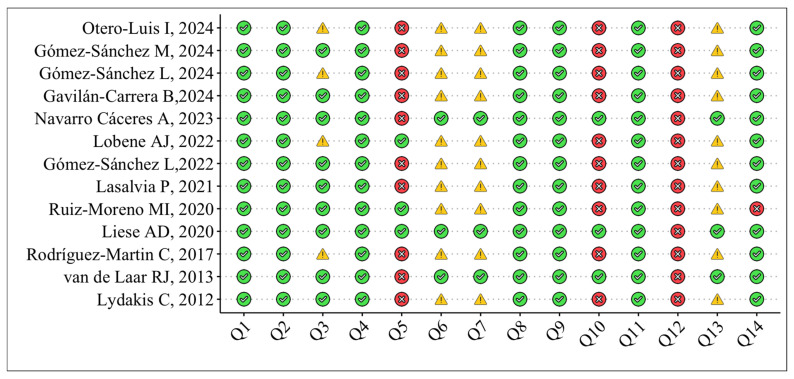
Quality assessment across selected studies [16,17,18,19,21,22,23,24,25,26,29,30,31].

Methods for assessing adherence to the Mediterranean diet showed heterogeneity among the selected studies: adherence scores such as the Mediterranean Diet Adherence Screener (MEDAS-14) [16,17,30], the Prevención con Dieta Mediterránea (PREDIMED) [18], the Mediterranean Diet Score [19], the Modified Mediterranean Diet Quality Index (mKIDMED) [21], and the Alternative Mediterranean Diet Score (aMED) [26,31]; adherence questionnaires such as the Mediterranean Diet Adherence Questionnaire (MD) [23], and the 14-item Food Consumption Frequency Questionnaire [29]; adherence indices such as the KIDMED Index (Mediterranean Diet Quality Index in Children and Adolescents) [24]. In addition, reports using the EVIDENT diet index were also included [22,27], as it is a good predictor of adherence to MD [22] (Figure 2).

As for the outcome measures, a series of markers related to arterial stiffness were considered, including aortic pulse wave velocity (a-PWv), carotid-femoral pulse wave velocity (cfPWV), brachial-ankle pulse wave velocity (baPWV), augmentation index (AIx, %) in the brachial artery, the distensibility coefficient (DC, a measurement of the elastic properties of arteries), and the cardio-ankle vascular index (CAVI).

Nine studies [16,17,19,22,23,25,29,30,31] reported the association between adherence to the Mediterranean diet and PWV values. Navarro Cáceres and colleagues [30], in the context of the EVA study, ran a multiple regression analysis between cfPWV and baPWV and the total score of the Mediterranean diet, finding a negative association (β −0.025; 95% CI −0.107 to 0.058, and β −0.064; 95% CI −0.131 to 0.003, respectively), but lacking significance. Lobene and colleagues [31], in a population of healthy American adults, carried out multiple regression analysis between aMED score and PWV, finding no association. Lasalvia and colleagues [25], in the context of the RoCAV (Risk of cardiovascular disease and abdominal aortic aneurysm in Varese) study, examined adherence to a Mediterranean-type dietary pattern in relation to cfPWV values (−0.18, 95% CI: −0.36 to 0.004; *p* = 0.06), and no significant association was found. Gómez-Sánchez and colleagues [23], in the context of the MARK Study (intermediate cardiovascular risk), studied adherence to a Mediterranean-type dietary pattern in relation to baPW values and found that there was a decrease of −0.052 (95%CI −0.141 to −0.008) in baPW. The same researchers [17], using data from the EVA, MARK, and EVIDENT studies, found an inverse relationship between BaPWV (m/s) and the Mediterranean diet (β −0.126, 95% CI −0.164 to −0.089, *p* < 0.001). Otero-Luis et al. [16], within the framework of the EvasCu trial, discovered that patients exhibiting low adherence demonstrated reduced a-PWV values compared to those with good adherence to MD (5.91 ± 1.33 m/s vs. 6.63 ± 1.27 m/s) (unadjusted model, *p* < 0.001). When the analyses were adjusted, no significant differences in a-PWV were shown according to the categories of adherence to the MD (*p* = 0.695). Rodríguez-Martin and colleagues [22], in the context of a Spanish population of the EVIDENT study, studied multiple regression analysis between the EVIDENT diet index and PWV, finding an inverse and significant relationship (β −0.089, 95% CI −0.148 to −0.030, *p* = 0.003). Gavilán-Carrera and colleagues [19] found no association between overall adherence to the Mediterranean diet and PWV (*p* > 0.05) in a population of female patients with systemic lupus erythematosus (SLE). Likewise, no correlation between cfPWV values and adherence to the Mediterranean diet was found in the analyses conducted by Ruiz-Moreno and colleagues [29].

Seven studies [20,21,24,27,28,30,31] examined the correlation between adherence to the Mediterranean diet and the AIx, a metric of systemic arterial stiffness obtained from the ascending aortic pressure waveform, revealing a statistically significant adverse relationship. Lobene and colleagues [31] ran a multiple regression analysis and found that the aMED score was inversely associated with AIx (β−1.59, 95% CI −3.09 to −0.09, *p* = 0.04). Navarro Cáceres and colleagues [30] ran a multiple regression analysis of CAIx and the total Mediterranean diet score, finding a negative, not significant association (β−0.242; CI 95% −0.358 to 0.843). Lee and colleagues [28] conducted a mixed models analysis of variance (ANOVA) to analyze the effect of the Mediterranean diet intervention on Aix in healthy Australian women, finding a significant decrease after the dietary intervention (*p* = 0.02). Recio-Rodríguez and colleagues [27] performed the ANCOVA model to analyze the effect of the intervention on the Mediterranean diet, finding a post-intervention decrease in the PAIx75 group at 3 months (mean difference −4.9%; 95% CI −7.7 to −2.1) and 12 months (mean difference −3.9%; 95% CI −6.8 to −1.0). Lydakis and colleagues [24] performed a multiple linear regression analysis between the KIDMED index and AIx, finding an inverse coefficient (β −0.114) and a statistically significant association (*p* = 0.026) in a population of high school children. Liese and colleagues [21] analyzed the relationship between diet quality and AIx, finding an inverse association (linear regression model between the mKIDMED and AIx: β −0.279). Jennings and colleagues [20] performed ANCOVA models between AIx and the Mediterranean diet. *p* value for the difference between the intervention and control groups, finding a significant inverse association (β −6.1, 95% CI, −12.5 to 0.3, *p* = 0.04).

Two studies [17,30] analyzed the association between the score for adherence to the Mediterranean diet and the cardio-ankle vascular index (CAVI), an index of the overall stiffness of the artery from the origin of the aorta to the ankle, finding a statistically significant inverse association. In fact, Gómez-Sánchez and colleagues [17] conducted a multiple regression analysis between CAVI and Mediterranean diet. They found a significant inverse relationship (β −0.045, 95% CI −0.062 to −0.028, *p* < 0.001), as did Navarro Cáceres and colleagues [30] (β −0.051; 95% CI −0.092 to −0.010, significant: *p* = 0.016).

Lastly, van de Laar and colleagues [26] compared longitudinal levels of adherence to a Mediterranean dietary pattern (aMED score) during adolescence and adulthood (two to eight repeated measurements obtained between 13 and 36 years of age) among individuals with different levels of arterial stiffness in adulthood, and found that individuals with stiffer carotid arteries (defined based on the most adverse tertile of the distensibility coefficient) had lower aMED scores (−0.32, 95% CI −0.6 to −0.06) and were less likely to have adhered to this dietary pattern (aMED score ≥ 5, odds ratio 0.69, 95% CI 0.50 to −0.94) during the preceding 24 years compared with those with less stiff arteries.

A single study [26], in the context of The Amsterdam Growth and Health Longitudinal Study, conducted generalized estimating equations (GEE) to study the average differences in aMED score during the 24-year longitudinal period: individuals with stiffer carotid arteries (defined based on the most unfavorable tertile of the distensibility coefficient) had lower aMED scores (−0.32, 95% CI −0.60 to −0.06) and were less likely to have adhered to this dietary pattern (aMED score ≥ 5, odds ratio 0.69, 95% CI 0.50 to −0.94) during the previous 24 years.

The quality assessment of the selected studies was rated as moderate to high, using the National Institutes of Health quality assessment toolkits for observational cohort and cross-sectional studies. The risk was rated as high for domains 5, 10, and 12, related to sample size justification, repeated exposure measurement (not applicable to cross-sectional reports), and blinded assessors.

## 4. Discussion

The present systematic review addressed the conceptual hypothesis of a link between adherence to a Mediterranean dietary pattern, assessed by validated adherence scores or scales, and stiffness of the arteries, assessed by a series of markers including aortic pulse wave velocity (a-PWv), carotid-femoral pulse wave velocity (cfPWV), brachial-ankle pulse wave velocity (baPWV), augmentation index (AIx, %) in the brachial artery, the distensibility coefficient (DC, a measurement of the elastic properties of arteries), and the cardio-ankle vascular index (CAVI). Therefore, a systematic review of existing observational studies and RCTs exploring the association between the Mediterranean diet and arterial stiffness was provided, concluding that there is an inverse relationship in the association, consistent in all selected studies (except for three reports that found no association [19,29,31]), regardless of the outcome measure considered as a proxy for stiffness. As outcomes, the selected studies reported a greater amount of evidence regarding PWV, the distance traveled by blood flow divided by the time it takes to travel that distance (m/s), and AIx, a measure of systemic arterial stiffness derived from the ascending aortic pressure waveform. However, a subset of the evidence showing an inverse relationship between adherence to the Mediterranean diet and PWV lacked significance [25,30], whereas the body of evidence showing an inverse association between adherence to the Mediterranean pattern and AIx values consistently reached statistical significance and benefited from the RCT design of three [20,27,28] of the seven studies. Less [17,30] but consistent and statistically significant evidence was also found in the cross-tabulation of adherence to the Mediterranean diet and the cardiovascular ankle index (CAVI), a proxy of the overall stiffness of the artery from the origin of the aorta to the ankle.

Over the decades, the Mediterranean diet, characterized by a high intake of fruits, vegetables, whole grains, legumes, nuts, seeds, fish, and healthy fats (especially olive oil) [32], has been scientifically proven to have several beneficial effects on vascular function [32,33,34]. These latter stem from several interrelated biological mechanisms that promote vascular function and reduce the age-related stiffening of arteries. One of the primary mechanisms is the reduction of inflammation. Chronic low-grade inflammation plays a pivotal role in the pathogenesis of arterial stiffness. The Mediterranean diet, rich in anti-inflammatory foods such as fruits, vegetables, nuts, legumes, and particularly extra virgin olive oil, has been shown to reduce the levels of inflammatory biomarkers like C-reactive protein (CRP) and interleukins (TNF-α, IL-6). Polyphenols, abundant in olive oil and other plant-based foods, act as potent anti-inflammatory agents by modulating immune cell function and reducing the expression of inflammatory cytokines. Studies suggest that adherence to the Mediterranean diet results in lower circulating levels of these inflammatory markers, which in turn contributes to better endothelial function and reduced arterial stiffness [35,36]. Moreover, omega-9 fatty acids, predominantly present in olive oil, are vital in enhancing cardiovascular health by lowering LDL cholesterol levels and optimizing vascular function. They mitigate inflammation and oxidative stress, both associated with cardiovascular disease. The habitual intake of olive oil correlates with a diminished risk of cardiovascular disease and cerebrovascular accidents.

Another key biological pathway through which the Mediterranean diet influences arterial stiffness is its antioxidant properties. Oxidative stress, which occurs when there is an excess of reactive oxygen species (ROS) and insufficient antioxidant defense, is a major contributor to endothelial dysfunction and the development of arterial stiffness. The Mediterranean diet, rich in antioxidants from foods such as fruits, vegetables, and particularly olive oil, helps neutralize ROS. These antioxidants include polyphenols, flavonoids, and vitamins C and E, all of which play a role in protecting the endothelium from oxidative damage. Extra virgin olive oil, a hallmark of the Mediterranean diet, contains phenolic compounds that reduce oxidative stress, improve endothelial function, and preserve vascular elasticity. Additionally, the consumption of polyphenol-rich foods like berries and vegetables has been linked to lower levels of oxidative stress and improved vascular health. By mitigating oxidative damage, the Mediterranean diet helps maintain the flexibility of arteries, leading to lower PWV and AIx [37].

The Mediterranean diet is also known to improve lipid profiles, another factor contributing to reduced arterial stiffness. A healthy lipid profile, characterized by high levels of high-density lipoprotein (HDL) cholesterol and low levels of low-density lipoprotein (LDL) cholesterol, is crucial for vascular health [1,33]. The Mediterranean diet has been shown to increase HDL cholesterol, which has protective effects on the endothelium, and decrease LDL cholesterol, a major contributor to atherosclerosis and arterial stiffening. Reducing triglycerides further improves the lipid profile, reducing the risk of plaque formation and subsequent arterial stiffness.

Blood pressure regulation is another significant path by which the Mediterranean diet reduces arterial stiffness [38,39]. High blood pressure is a well-known risk factor for arterial stiffening, and the Mediterranean diet has been shown to have beneficial effects on blood pressure, mainly through the consumption of potassium- and magnesium-rich foods like fruits, vegetables, and nuts. These minerals help to relax blood vessels and lower blood pressure, which alleviates the mechanical stress on arterial walls. Additionally, omega-3 fatty acids, found in fatty fish and nuts, also help reduce blood pressure and improve endothelial function [40,41].

The Mediterranean diet also improves endothelial function through the enhanced production of nitric oxide (NO) [42]. Nitric oxide is a vasodilator that plays a crucial role in maintaining arterial elasticity. It is well-acknowledged that a diet rich in antioxidants, polyphenols, and healthy fats, like those found in fruits, vegetables, and olive oil, can enhance nitric oxide production and improve endothelial function. These nutrients reduce oxidative stress and inflammation, promoting better blood vessel health and circulation. The polyphenols and omega-3 fatty acids found in the Mediterranean diet promote the production of nitric oxide by increasing the activity of endothelial nitric oxide synthase (eNOS). This leads to improved vascular tone, reduced arterial stiffness, and better overall vascular health. The ability of the Mediterranean diet to enhance endothelial function through increased nitric oxide production is a significant factor in reducing arterial stiffness [43,44].

Also, emerging evidence also suggests that the Mediterranean diet may influence arterial stiffness through its effects on the gut microbiome [45,46]. A healthy gut microbiota, which is promoted by the high fiber content of the Mediterranean diet, plays an important role in regulating systemic inflammation, lipid metabolism, and even blood pressure. Short-chain fatty acids (SCFAs), produced by gut bacteria during the fermentation of dietary fibers, have been shown to reduce inflammation and improve endothelial function, potentially contributing to the reduction of arterial stiffness. While this area of research is still developing, it provides an additional layer of understanding regarding the Mediterranean diet’s vascular benefits.

Lastly, foods commonly included in the Mediterranean diet, such as fatty fish, fortified dairy products, and egg yolks, are rich in vitamin D and can support endothelial function, reduce inflammation, and promote overall cardiovascular health.

Altogether, the beneficial effects of the Mediterranean diet on arterial stiffness are the result of a combination of mechanisms, including anti-inflammatory effects, antioxidant properties, improvement of lipid profiles, regulation of blood pressure, improvement of endothelial function, and, potentially, modulation of the gut microbiome. These factors collectively contribute to reduced vascular stiffness, as evidenced by the improvement in PWV and AIx, among other arterial stiffness parameters. While research continues to explore the specific pathways through which the Mediterranean diet influences vascular health, it is clear that this diet plays a fundamental role in preserving arterial flexibility and reducing the risk of cardiovascular disease.

To sum up, a comparison between the cardioprotective potential of the Mediterranean diet and other dietary models, such as the DASH (Dietary Approaches to Stop Hypertension) diet and the Nordic diet, gives a contextualized perspective. The Mediterranean diet is widely recognized for its cardiovascular benefits thanks to the high content of healthy fats, particularly olive oil, fruits, vegetables, fish, and legumes, which reduce inflammation and improve endothelial function. The DASH diet, which focuses on reducing blood pressure, emphasizes foods that are low in sodium and rich in potassium, calcium, and magnesium, which have a strong impact on the prevention of hypertension and cardiovascular health. The Nordic diet, similar to the Mediterranean diet, includes foods rich in omega-3 fatty acids from fish and seeds but emphasizes typical northern European products such as whole grains, root vegetables, and green leafy vegetables. Overall, all three diets show cardioprotective potential, but the Mediterranean diet is the most studied for its association with reducing the risk of cardiovascular disease, while the DASH diet is particularly effective in controlling blood pressure. The Nordic diet, although beneficial, is less well-known in terms of its long-term effects on heart health than the other two.

### Limitations

Our findings should be considered in the context of many limitations. At first, the observational nature of the included studies, especially those employing a cross-sectional design, may lead to reported relationships being affected by residual or unmeasured confounding, despite the use of numerous variables. Moreover, the utilization of the FFQ for evaluating daily food consumption, while a prevalent and proven approach in epidemiological studies, is subject to reporting bias associated with self-assessed intake. This comprehensive study enhances the research on the Mediterranean diet and arterial stiffness as a cardiovascular risk factor, which previously lacked a qualitative synthesis. However, the findings must be interpreted cautiously due to the inherent constraints previously outlined.

## 5. Conclusions

A qualitative synthesis of the available evidence provides consistent proof that people exposed to a Mediterranean diet may have less stiff arteries and therefore a lower cardiovascular risk, most likely through a multifactorial biological pathway.

## Figures and Tables

**Table 1:** The search method utilized for the US National Library of Medicine (PubMed) and the Medical Literature Analysis and Retrieval System Online (MEDLINE) and modified for other sources based on chosen descriptors.

	Strategy	Descriptors Used
#1	Population type	(human*[tiab])
#2	Exposure	(mediterranean diet*[tiab]) OR (mediterranean pattern*[tiab])
#3	Outcome	(arterial stiffness[tiab]) OR (carotid-femoral pulse wave velocity[tiab]) OR (pulse wave velocity[tiab]) OR (augmentation index[tiab]) OR (Pulse Wave Velocity[tiab])
#4	Exclusion keywords	(Review[tiab]) OR (systematic review[tiab]) OR (narrative review[tiab]) OR (meta-analysis[tiab]) OR (editorial[tiab]) OR (letter[tiab]) OR (commentary[tiab]) OR (perspective[tiab]) OR (book[tiab])
#5	Search strategy	#1 AND #2 AND #3 NOT #4
	Filters: Sort by: Most Recent. Date: 31 December 2024. Species: Humans. Time restriction: none.	

**Table 2:** Descriptive of selected studies. *N* = 16.

Author, Year	Design	Country	Age	Sex	Intervention Duration	Exclusion Criteria	N	Population	Exposure Assessment Tool	Outcome	Strength of Association
Otero-Luis I, 2024 [16]	Cross-sectional	Spain (Europe)	18+ years	143M, 243F	NA	A diagnosis of diabetes mellitus, arterial hypertension, cancer, acute myocardial infarction, angina pectoris, chronic kidney disease, chronic obstructive pulmonary disease, or hypercholesterolemia.	386	EvasCu Study (healthy adults)	Mediterranean Diet Adherence Screener (MEDAS-14)	a-PWv	Adjusted (age, pulse pressure, body mass index, and educational level) differences in a-PWv (m/s) and adherence to the Mediterranean diet according to Student’s *t* test and ANCOVA: *p* = 0.695
Gómez-Sánchez L, 2024 [17]	Cross-sectional	Spain (Europe)	35 to 75 years	249M, 252F	NA	Having a terminal illness, inability to travel to the research unit, a history of cardiovascular disease, glomerular filtration rate < 30 mL/min/1.73 m^2^, chronic inflammatory disease or recent acute inflammation (within 3 months), or treatment with estrogen, testosterone, or growth hormone.	501	EVA Study	PREDIMED questionnaire	cfPWV	Age-adjusted Pearson correlation between cfPWV (m/s) and MD score: −0.082
Gómez-Sánchez M, 2024 [18]	Cross-sectional	Germany (Europe)	35 to 75 years	1943M, 1458F	NA	No cardiovascular disease (EVA study), intermediate cardiovascular risk (MARK study), known coronary or cerebrovascular disease, heart failure, moderate/severe COPD, musculoskeletal disease limiting walking, advanced respiratory/renal/hepatic disease, severe mental illness, or treated cancer within the last 5 years (EVIDENT study).	3401	EVA, MARK, and EVIDENT studies	MEDAS questionnaire	baPWV CAVI	Multiple regression analysis between BaPWV (m/s) and Mediterranean diet: β −0.126, CI 95% −0.164 to −0.089, *p* < 0.001 Multiple regression analysis between CAVI and Mediterranean diet: β −0.045, 95% CI −0.062 to −0.028, *p* < 0.001
Gavilán-Carrera B, 2024 [19]	Cross-sectional	Spain (Europe)	18 to 60 years	76F	NA	(i) Inability to sign the informed consent, (ii) clinical CV pathology in the past year, and (iii) prednisone doses > 10 mg/day in the last 6 months.	76	Female patients with systemic lupus erythematosus (SLE)	Mediterranean Diet Score	PWV	No association of the overall adherence to the Mediterranean Diet with PWV was found (all *p* > 0.05).
Jennings A, 2019 [20]	RCT	5 European centers (Bologna in Italy, Norwich in the United Kingdom, Wageningen in the Netherlands, Warsaw in Poland, and Clermont-Ferrand in France)	65 to 79 years	NR	1 year	Clinically diagnosed chronic disease, corticosteroid or insulin use, recent antibiotic/vaccination use, medication changes in the last three months, food allergies/intolerances requiring special diets, frailty (Fried criteria 1), or malnutrition.(defined as BMI < 18.5 kg/m^2^ or >10% weight loss in the previous six months).	225	The NU-AGE study (New Dietary Strategies Addressing the Specific Needs of Elderly Population for Healthy Aging in Europe)	NU-AGE index	PWV AIx	ANCOVA models between AIx and Mediterranean diet *p* value for difference between intervention and control groups: β −6.1 (95% CI, –12.5 to 0.3), *p* = 0.04
Liese AD, 2020 [21]	Longitudinal	USA	10 to 30 years	260M, 260F	7.7 years	Missing FFQ, not having T1D.	520	SEARCH for Diabetes in Youth Study (type 1 diabetes young population)	modified Mediterranean Diet Quality Index (mKIDMED)	PWV AIx	Linear regression model between the mKIDMED and AIx: β −0.2791
Rodríguez-Martin C, 2017 [22]	Cross-sectional	Spain (Europe)	20–80 years	936F, 617M	NA	Coronary or cerebrovascular disease, heart failure, moderate to severe COPD, musculoskeletal disease preventing walking, advanced liver/lung/kidney disease, severe mental illness, cancer treated or diagnosed within 5 years, end-stage disease, and pregnancy.	1553	EVIDENT study (no cardiovascular disease)	EVIDENT diet index	PWV	Multiple regression analysis between EVIDENT diet index and PWV: β −0.089, 95%CI −0.148 to −0.030, *p* = 0.003
Gómez-Sánchez L,2022 [23]	Cross-sectional	Spain (Europe)	35–75 years	1524M, 951F	NA	Presenting a disease in the terminal stage, being institutionalized at the time of the visit, or having a history of atherosclerosis.	2475	MARK Study (intermediate cardiovascular risk)	Mediterranean diet (MD) adherence questionnaire	baPWV	Multiple linear regression analysis between MD adherence score and baPWV: β −0.052 (95%CI −0141–−0.008)
Lydakis C, 2012 [24]	Cross-sectional	Greece (Europe)	12-year-old children	132M, 145F	NA	Unwillingness to consent, treated cardiac or renal conditions, diabetes mellitus, or use of immunosuppressive or cytotoxic drugs.	277	High school children	KIDMED index (Mediterranean Diet Quality Index in children and adolescents)	AIx	Multiple linear regression analysis between KIDMED index and AIx: β −0.114, *p* = 0.026
Lasalvia P, 2021 [25]	Cross-sectional	Italy (Europe)	men (50–75 years old) and women (60–75 years old)	1608M, 1032F	NA	Having main chronic diseases (diabetes, myocardial infarction, stroke, cancer), whose diagnosis could have modified the own dietary habit.	2640	RoCAV (Risk of Cardiovascular diseases and abdominal aortic Aneurysm in Varese) study	Mediterranean diet adherence score (MedS)	cfPWV	Linear regression model between a Mediterranean-like diet pattern and cfPWV: β −0.18 (95% CI −0.36, 0.01), *p* = 0.05
van de Laar RJ, 2013 [26]	Longitudinal	Netherlands (Europe)	13 to 36 years	196F, 177M	24 years	Lack of measurements of lifestyle (including dietary, physical activity, and smoking habits), anthropometric (height, weight, and skinfold measurements), and biological (BP and blood lipid levels).	373	The Amsterdam Growth and Health Longitudinal Study	aMED score	Distensibility coefficient	Generalized estimating equations (GEEs) to investigate the mean differences in the aMED score throughout the 24-year longitudinal period: individuals with stiffer carotid arteries (defined on the basis of the most adverse tertile of the distensibility coefficient) had lower aMED scores (−0.32, 95% CI −0.60; −0.06) and were less likely to have adhered to this dietary pattern (aMED score ≥ 5, odds ratio 0.69, 95% CI 0.50; −0.94) during the preceding 24 years
Recio-Rodríguez JI, 2019 [27]	RCT	Spain (Europe)	under 70 years	249F, 348M	1 year	History of cardiovascular events, musculoskeletal disease that limits walking, severe respiratory, renal or hepatic disease, a diagnosis of oncological processes in the last 5 years, and pregnant women.	597	EVIDENT II Study	EVIDENT application	PAIx75 CAVI baPWV	ANCOVA model to analyze the effect of the Mediterranean diet intervention: post-intervention decreases in the PAIx75 group at 3 months (mean difference −4.9%; 95% CI −7.7 to −2.1) and 12 months (mean difference −3.9%; 95% CI −6.8 to −1.0)
Lee J, 2014 [28]	RCT	Australia	20 to 38 years	24F	10 days	Medical conditions, such as food allergies, diabetes, or heart disease, as well as neurologic and psychiatric disorders. Individuals who were pregnant, breastfeeding, smoking, or had a history of substance abuse also were excluded.	24	Healthy women	Mediterranean-style eating plan	AIx	Mixed-model analysis of variance (ANOVA) to analyze the effect of the Mediterranean diet intervention on AIx: significant decrease post-intervention, *p* = 0.02
Ruiz-Moreno MI, 2020 [29]	Cross-sectional	Spain (Europe)	6–11 years	43M, 32F	NA	Participants who satisfied more than one criterion of metabolic syndrome, were outside the specified age range at baseline, or had diabetes or other metabolic disorders.	75	Subjects with obesity who had ≤1 metabolic syndrome criteria	14-item food consumption frequency questionnaire	cfPWV	Bivariate correlations using Pearson’s correlation coefficient: no correlation between cfPWV values and MedDiet adherence
Navarro Cáceres A, 2023 [30]	Longitudinal	Spain (Europe)	35–75 years	252F, 249M	5 years	Individuals with terminal illnesses, incapacity to access primary care facilities, a history of cardiovascular disorders, a glomerular filtration rate below 30%, chronic inflammatory diseases or acute inflammatory events within the past three months, or undergoing treatment with estrogen, testosterone, or growth hormone.	501	EVA study	14-item MEDAS questionnaire	cfPWV CAIx CAVI	Multiple regression analysis between cfPWV and total score of Mediterranean diet: negative association β −0.025; CI 95% −0.107 to 0.058, not significant Multiple regression analysis between baPWV and total score of Mediterranean diet: negative association β−0.064; CI 95% −0.131 to 0.003, not significant Multiple regression analysis between CAVI and total score of Mediterranean diet: negative association β−0.051; CI 95% −0.092 to −0.010, significant: *p* = 0.016 Multiple regression analysis between CAIx and total score of Mediterranean diet: negative association β−0.242; CI 95% −0.358 to 0.843, not significant
Lobene AJ, 2022 [31]	Cross-sectional	USA	18–45 years	34F, 22M	NA	A history of chronic disease (CVD, cancer, diabetes, kidney disease), use of related medications, BP ≥ 140/90 mmHg, BMI ≥ 30 kg/m^2^, tobacco use, or pregnancy/breastfeeding.	56	Healthy young adults	alternative Mediterranean Diet (aMED) score	PWV AIx	Multiple regression analysis between the aMED score and AIx: inverse association β −1.59, 95%CI −3.09 to −0.09, *p* = 0.04

Abbreviations: Aortic pulse wave velocity (a-PWv); Carotid–femoral pulse wave velocity (cfPWV); Brachial–ankle pulse wave velocity (baPWV); Cardio ankle vascular index (CAVI); Pulse wave velocity (PWV); Augmentation index (AIx); Brachial-ankle pulse wave velocity (baPWV); Peripheral augmentation index (PAIx75); Cardio-ankle vascular index (CAVI); Central augmentation index (CAIx).

## Data Availability

Any inquiry can be directed to the corresponding author.
